# Optimization of Polylactide-Co-Glycolide-Rifampicin Nanoparticle Synthesis, In Vitro Study of Mucoadhesion and Drug Release

**DOI:** 10.3390/polym16172466

**Published:** 2024-08-30

**Authors:** Nazgul A. Yessentayeva, Aldana R. Galiyeva, Arailym T. Daribay, Daniyar T. Sadyrbekov, Rouslan I. Moustafine, Yerkeblan M. Tazhbayev

**Affiliations:** 1 Chemistry Department, Karaganda Buketov University, Karaganda 100028, Kazakhstan; naz.yessentayeva92@gmail.com (N.A.Y.); arailymdaribay@gmail.com (A.T.D.); daniyar81@gmail.com (D.T.S.); 2 Institute of Pharmacy, Kazan State Medical University, Kazan 420126, Russia; ruslan.mustafin@kazangmu.ru

**Keywords:** nanoparticles, polylactide-co-glycolide, rifampicin, central composite design, tuberculosis

## Abstract

Despite the large number of works on the synthesis of polylactide-co-glycolide (PLGA) nanoparticles (NP) loaded with antituberculosis drugs, the data on the influence of various factors on the final characteristics of the complexes are quite contradictory. In the present study, a comprehensive analysis of the effect of multiple factors, including the molecular weight of PLGA, on the size and stability of nanoparticles, as well as the loading efficiency and release of the antituberculosis drug rifampicin (RIF), was carried out. Emulsification was carried out using different surfactants (polyvinyl alcohol, Tween 80 and Pluronic F127), different aqueous-to-organic phase ratios, and different solvents (dichloromethane, dimethyl sulfoxide, ethyl acetate). In this research, the PLGA nanoemulsion formation process was accompanied by ultrasonic dispersion, at different frequencies and durations of homogenization. The use of the central composite design method made it possible to select optimal conditions for the preparation of PLGA-RIF NPs (particle size 223 ± 2 nm, loading efficiency 67 ± 1%, nanoparticles yield 47 ± 2%). The release of rifampicin from PLGA NPs was studied for the first time using the flow cell method and vertical diffusion method on Franz cells at different pH levels, simulating the gastrointestinal tract. For the purpose of the possible inhalation administration of rifampicin immobilized in PLGA NPs, their mucoadhesion to mucin was studied, and a high degree of adhesion of polymeric nanoparticles to the mucosa was shown (more than 40% within 4 h). In the example of strain H37Rv in vitro, the sensitivity of *Mycobacterium tuberculosis* to PLGA-RIF NPs was proven by the complete inhibition of their growth.

## 1. Introduction

In order to develop drug delivery systems (DDS) for the transport and controlled release of drugs, polymeric nanoparticles based on biocompatible and biodegradable polymeric materials are increasingly being synthesized, with different advantages [1,2,3]. One of the most promising polymers that is often used for this purpose is PLGA. The copolymer of lactic and glycolic acid has unique properties, such as biocompatibility, biodegradability, and the ability to control the rate of drug release [4,5,6]. These characteristics make it an ideal material for creating drug carriers that are capable of delivering the drug to the body in a stable and controlled manner. This is critical for the effective treatment of a health-threatening disease such as tuberculosis (TB) [7,8], which claims 1.30 million lives worldwide each year, and many more are physically disabled by the disease [9,10]. Rifampicin has been used for many years to treat tuberculosis, and has been shown to be highly effective. RIF is highly lipophilic, which favors its penetration through cell membranes, and it is classified by the Biopharmaceuticals Classification System as a drug with low solubility but high permeability (Class II) [11]. RIF is administered orally to patients in relatively high concentrations, but it is taken in several doses throughout a day in case of poor tolerance. This causes not only toxic effects of RIF in the organism, but also often leads to acquired *Mycobacterium tuberculosis* (MTB) drug resistance after 1 month [12,13]. This problem can be solved by creating an inhaled dosage form of RIF [13,14]. Mucosal administration provides higher bioavailability and targeted delivery, allowing the drug to rapidly enter the bloodstream and concentrate directly in the lungs, which is particularly useful for the treatment of tuberculosis. The encapsulation of anti-TB drugs into PLGA NPs will increase their solubility in the lungs. Moreover, the necessary drug concentration in targeted alveolar macrophages can be achieved by controlling the particle size [14].

Over the past two decades, many methods of nanoparticle synthesis have been developed [15], but scientific attention is now increasingly focused on the optimization of different parameters to obtain effective compositions. Nanoparticle preparation methods have to be analyzed and adapted for each active ingredient. However, the average nanoparticle size and the drug loading efficiency play a key role in successful and efficient drug delivery to alveolar macrophages, especially in the development of delivery systems for inhalation applications [13,14]. For example, a particle size of 200–500 nm is considered optimal for effective drug delivery to alveolar macrophages, as the particle size significantly affects their absorption capacity [14,15,16]. In addition, it is worth noting that a number of studies have observed the low loading efficiency of rifampicin into the PLGA polymer matrix, highlighting the importance of addressing this issue in our work [17,18,19]. Therefore, the central composite design (CCD) method was chosen for the optimization. CCD can be used to evaluate nonlinear dependencies between process parameters, and therefore it is critical for the accurate modeling and prediction of nanoparticle characteristics. The center points of the experimental range and star points within CCD allow the curvature of the response surface to be determined, which helps to identify complex interactions and optimal conditions to produce nanoparticles with desired properties [20,21]. This method also provides maximum information with a minimum number of experiments, so it significantly reduces time and resources.

Due to the complexity of the optimization of PLGA NPs formation, it is important to find the optimal conditions for the synthesis of polymer nanoparticles with given physicochemical characteristics, and to evaluate drug release and the ability to inhibit the growth of MTB. In this regard, the most relevant aspect is the optimization of synthesis conditions, allowing us to produce PLGA NPs encapsulated by RIF with the given characteristics, to study the mucoadhesion of nanoparticles and to determine the drug release profile from the polymer under conditions approximating the conditions in the body, all of which helps us to consider the activity of this polymer–drug complex against MBT.

## 2. Materials and Methods

### 2.1. Materials

Poly (D,L-lactide-co-glycolide) (50:50)-ester with terminated molecular weight (MW) 7000–17,000, 24,000–38,000 and 30,000–60,000; Pluronic F-127 powder; olyvinyl alcohol (PVA) (MW 9000–10,000, 80% hydrolyzed); rifampicin with indicated purity over 99%; dimethyl sulfoxide (DMSO) (≥99.5); ethyl acetate (EA) (≥99.5); dichloromethane (DCM) (≥99.5); mucin from porcine stomach, Type II. All reagents were purchased from Sigma Aldrich (St. Louis, MO, USA). Tween 80 solution was purchased from CJSC “Kupavnareaktiv” (Russia, Old Kupavna city).

### 2.2. Preparation of Rifampicin Loaded PLGA Nanoparticles

PLGA nanoparticles containing rifampicin were prepared by the simple emulsion and solvent evaporation method [4,22]. The procedure can be described as follows: a given amount of rifampicin and PLGA was dissolved in an organic solvent (DMSO, DCM, or EA), after which this solution was emulsified for 1 min on an ultrasonic homogenizer (Bandelin Sonopuls HD 2070, Bandelin Elec., Berlin, Germany). Different surfactant concentrations were then added to the produced solution and homogenized on an ultrasonic homogenizer for 5, 10 or 15 min at a given power (15, 35 or 75 W). Finally, the prepared dispersion was subjected to magnetic stirring (400 rpm) to evaporate the organic solvent at room temperature overnight. After the evaporation of the solvent, the nanosuspension was centrifuged (Centrifuge 5420, Eppendorf, Hamburg, Germany) at 15,000 rpm for 20 min, then the produced nanoparticles were washed with distilled water (50 °C) to remove surfactant residues and dried at room temperature.

### 2.3. Experimental Design of Central Composite Design

The optimization of the production of RIF-encapsulated PLGA NPs was performed using central composite design by use of various factors such as polymer to drug ratio, aqueous to organic phase ratio, surfactant concentrations, and the duration and power of ultrasonic homogenization. In addition, different surfactants (PVA, Tween 80 or Pluronic F127) were used and DMSO, DCM and EA were tested as the organic phase. Design Expert^®^ software (version 13, Stat-Ease, Minneapolis, MN, USA) was used to create a CCD array of eight factors at three levels each (Table 1).

### 2.4. Measurement of Particle Size, Polydispersity and Zeta Potential

Nanoparticle diameter and polydispersity index (PDI) were measured using a Dynamic Light Scattering by Zetasizer Nano S90 device manufactured by Malvern Instruments Ltd., Malvern, UK. For this purpose, 5–8 drops of nanoparticle suspension were redispersed in 1.5–2 mL of distilled water. Analyses were carried out at 25 °C with 90° angle determination. The ζ-potential was determined with a ζ-potential analyzer (NanoBrook ZetaPALS, Holtsville, NY, USA) using electrophoretic laser Doppler anemometry. In addition, scanning electron microscopy (SEM) (MIRA 3LMTESCAN, Brno, Czech Republic, EU) was used to investigate the size, shape and surface morphology of the nanoparticles. For the experiment, the samples were coated with conductive coatings using carbon rods for SEM. The stub type for the electron microscope was made of aluminum alloy. The SEM scanning conditions were as follows: accelerating voltage—HV 5 kV, working distance—WD 5.14 mm, magnification—115,000 kx.

### 2.5. Determination of Drug Loading Efficiency and Nanoparticle Yield

To determine the amount of drug encapsulated in the polymer matrix, the mass of unencapsulated drug in the supernatant was calculated. For this purpose, high-performance liquid chromatography (HPLC) using Shimadzu LC-20 Prominence and UV/VIS detector (Shimadzu Scientific Instruments, Kyoto, Japan) equipment at 254 nm wavelength was undertaken [23]. Acetonitrile–water eluent (60–40) at a flow rate of 0.8 mL/min through a Promosil C18 column (Agilent Technologies, Tokyo, Japan) (sorbent grain size 5 μm, 100 Å, 4.6 × 150 mm) was used for separation of the components. The column temperature was maintained at 40 °C. Quantitative analyses were performed using a calibration curve. The injection volume was 10 μL (loop injection). The loading efficiency was calculated according to the formula below:

$$Loading\;efficiency\;\left(LE,\;\%\right)=\frac{Total\;mass\;of\;RIF-mass\;of\;freeRIF}{Mass\;of\;NPs}\times 100\%$$

$$Nanoparticles\;yield\;\left(\%\right)=\frac{Mass\;of\;NPs}{Total\;mass\;of\;RIF+mass\;of\;PLGA}\times 100\%$$

### 2.6. In Vitro Release of Drug from Polymer Nanoparticles

Two methods were used to evaluate drug release in vitro: the Flow-Through Cell method on a CE 7Smart (Sotax, Aesch, Switzerland) and the vertical diffusion method on a Franz PHOENIX™ DB-6 cell (Telodyne Hanson Research, Chatsworth, GA, USA) [24,25]. Experiments were performed at 37 ± 0.5 °C using phosphate buffer solutions of three selected pH values to mimic human body conditions: pH 1.2 (ionic strength 0.01 M) to mimic the gastric environment; pH 6.8 (ionic strength 0.21 M) to mimic the intestinal environment; and pH 7.4 (ionic strength 0.26 M) to mimic blood plasma pH.

In the process of performing the flow cell method on a CE 7Smart instrument, the flow rate of the solution was maintained at 2 mL/min in a closed-loop configuration, utilizing a dialysis membrane featuring pores with a diameter ranging from 8000 to 10,000 Da in the form of Float-A-Lyzer^®^ G2 nanoadapters (MerckKGaA, Darmstadt, Germany). The duration of the experiment was 48 h.

Utilizing the vertical diffusion approach within a Franz cell setup, polymeric nanoparticles containing an anti-TB agent were positioned on the surface of a dialysis membrane (MWCO = 8000 Da, Medicell International Ltd., London, UK) while maintaining a mixer speed of 200 rpm. The experiment was sustained for a period of 48 h.

Every 0.5, 1, 2, 4, 8, 24 and 48 h, portions of the medium were extracted and analyzed using a UV/Vis spectrophotometer Lambda 25 (PerkinElmer, Waltham, MA, USA) at 475 nm. Subsequently, the extracted portions were reintroduced. The findings were then systematically juxtaposed with the calibration curve derived from standard solutions with various known concentrations. The rate of drug release was determined by the subsequent equation.

The amount of released drug was calculated using the formula below,

$$Drug\;release\left(\%\right)=\frac{Mass\;of\;released\;RIF}{Mass\;of\;total\;RIF\;in\;nanoparticles}\times 100\%$$

### 2.7. Thermogravimetric Analysis and Differential Scanning Calorimetry

Thermogravimetric and differential scanning calorimetry analyses were performed on a LabSYS evo TGA/DTA/DSC analyzer from Setaram (Caluire, France). The temperature spectrum was varied from 30 to 550 °C. The substance was heated in an aluminum oxide crucible. The sample was heated at 10 °C/min in a nitrogen atmosphere at a flow rate of 30 mL/min.

### 2.8. Study of Prepared Nanoparticles by Infrared Spectroscopy

The samples were analyzed using infrared spectroscopy with the FSM 1202 spectrometer from Infraspek Ltd. (Saint Petersburg, Russia). Fourier-transform infrared (FTIR) spectra were obtained using the KBr method. A pellet was prepared by blending around 3 mg of the sample with 100 mg of KBr. The scanning range was set from 4000 to 500 cm^−1^, with a resolution of 8 cm^−1^.

### 2.9. In Vitro Study of Nanoparticle Mucoadhesion

To evaluate the mucoadhesive properties of nanoparticles in vitro, a turbidimetric method was used. For this purpose, the absorbance of PLGA-RIF NPs with mucin dispersion was measured at 258 nm on a UV–visible spectrophotometer. Mucin in phosphate buffer solution at pH 6.4 (with a concentration of 0.125 mg/mL) and nanoparticles were mixed and incubated at 37 °C with constant stirring for 1, 2, 3 and 4 h [26]. Before starting each experiment, the turbidity of the NPs solution was determined as a baseline. The interaction of mucin and PLGA-RIF NPs was calculated using the following equation,

$$Mucin\;binding\;efficiency\;\left(\%\right)=\frac{Initial\;mucin\;\left(amount\right)-Free\;mucin\;\left(amount\right)}{Initial\;mucin\;\left(amount\right)}\cdot 100\%$$

### 2.10. Statistical Processing of the Produced Data

All studies were carried out at least three times. The results are presented as mean value with standard deviation. In order to compare the independent groups, the results were analyzed by one-way ANOVA followed by Tukey’s test, performed using Minitab 19 software, and a *p*-value < 0.05 was accepted as significant. The equation used for the calculation of percentage prediction error [27] was

$$Error\;\left(\%\right)=\frac{Predicted-Experimental}{Experimental}\;\cdot 100\%$$

## 3. Results and Discussion

### 3.1. Synthesis and Optimization of Conditions for Producing PLGA-RIF NPs

The synthesis of PLGA NP was carried out by the emulsification of organic polymer solution into an aqueous solution in the presence of a surfactant followed by solvent evaporation. DMSO, DCM and EA, in which RIF dissolves well, were used as organic solvents. According to various sources, the characteristics of PLGA NPs are different depending on the nature of the surfactant, so in this study we attempted to use the most commonly used surfactants in the pharmaceutical industry, including Tween 80, PVA and Pluronic F127. In most of the analyzed works, one of the problems considered is the difficulty of producing relatively homogeneous particles with a diameter of less than 200 nm [28,29,30]. However, none of them mention the use of an ultrasonic homogenizer to regulate the characteristics of PLGA nanoparticles. In this study, we used ultrasonic dispersion on a Bandelin Sonopuls HD 2070 homogenizer (Bandelin Elec., Germany) to form a homogeneous emulsion. The power and duration of ultrasonic homogenization were chosen as the optimization parameters. Another little-studied factor that is potentially significant in the synthesis of PLGA NPs is the molecular weight of the polymer. Using neural networks, it was predicted that the size of the particles formed will increase with the growth of the polymer MW, but the PDI will not change linearly according to the calculations [31]. To refine this prediction, we also considered the effect of PLGA molecular weight on the significant physicochemical characteristics of NPs, such as nanoparticle diameter, polydispersity, surface charge and drug loading efficiency.

To comprehensively optimize the above synthesis parameters, we applied the central composite design method. This optimization method is known to determine the interactive effect of a large number of variables affecting the results/product quality by performing a limited number of experiments. Previously, the CCD method has shown good and reliable predictions in the optimization of the synthesis of Human Serum Albumin NPs, but with a much smaller number of parameters [32].

Table 2 shows the results of the influence of the estimated variables on the drug loading efficiency and average particle size. According to the data obtained, depending on the synthesis conditions, PLGA particles with diameters ranging from 93 ± 2 to 452 ± 3 nm can be produced, while the polydispersity index also varies significantly from 0.046 ± 0.003 to 0.556 ± 0.039. NPs with the smallest diameter can be synthesized under conditions corresponding to NP35 in Table 2. However, in this case, the drug loading efficiency and the nanoparticles yield have minimum values, which is undesirable. As far as the value of drug loading efficiency and nanoparticle yield are concerned, they vary very significantly from 4% to 88.2% and 3.4% to 75.2%, respectively. The values of zeta potentials of nanoparticles, on the other hand, vary from –30.9 ± 3.4 to –0.61 ± 2.1 mV. The lowest value of zeta potential was recorded for NP1, showing that the system is stable in terms of electrostatic interaction, as charged particles repel each other to minimize the probability of aggregation [30,33].

PLGA30 is for the poly (D,L-lactide-co-glycolide) (50:50)-ester terminated with molecular weight 30,000–60,000. Analysis of variance (ANOVA) was used to assess the suitability and significance of the mathematical model used in predicting particle size and loading efficiency (see Table 3). The multiple regression results indicate that quadratic terms should be considered in the mathematical model for the reliable determination of responses.

Based on the data presented in Table 3, a *p*-value for both responses (mean NPs size and drug loading efficiency) below 0.05 confirms the significance of the model conditions. For the mean NPs size, the model F-value of 3.33 indicates the significance of the model with only 0.71% probability of noise occurrence. Similarly, the model F-value of 8.98 for the drug loading efficiency emphasizes its significance with only 0.01% probability of occurrence due to noise.

The model created using CCD to estimate the particle size and loading efficiency is shown below. In these formulas: A—PLGA:RIF ratio, B—MW of PLGA, C—surfactant, D—concentration of surfactant, E—organic solvent, F—homogenization power, G—homogenization time, H—organic phase:aqueous phase ratio.

Size = 207.83 + 40.46A + 3.61B + 1.68C + 5.68D − 30.75E − 19.91F + 7.94G + 10.57H + 8.52AB − 10.64AC + 4.71AD − 5.18AE − 24.24AF + 18.82AG + 13.41AH − 2.55BC + 7.73BD − 7.93BE − 8.34BF + 0.1857BG + 12.66BH + 0.0634CD − 2.56CE − 0.4044CF + 12.64CG + 20.98CH + 4.23DE − 7.49DF + 12.88DG + 8.46DH + 29.07EF − 2.91EG + 48.13EH − 15.09FG + 8.38FH − 14.40GH − 33.54A^2^ − 9.29B^2^ − 8.79C^2^ + 47.71D^2^ + 78.96E^2^ − 14.79F^2^ − 35.19G^2^ + 24.01 H^2^

Drug loading = 37.23 + 4.21A − 0.3393B + 0.6190C − 2.56D − 4.46E + 6.99F − 3.05G − 16.49H + 1.76AB + 0.6751AC + 4.76AD + 4.70AE − 5.55AF − 1.31AG + 6.12AH − 1.35BC + 2.66BD − 3.65BE − 2.81BF + 2.23BG + 3.76BH + 2.36CD − 1.53CE + 4.78CF + 1.29CG − 0.6921CH − 1.33DE − 2.73DF + 1.06DG − 1.49DH + 4.83EF − 6.05EG − 2.47EH + 1.12FG − 1.23FH + 2.54GH

**Figure 1:**
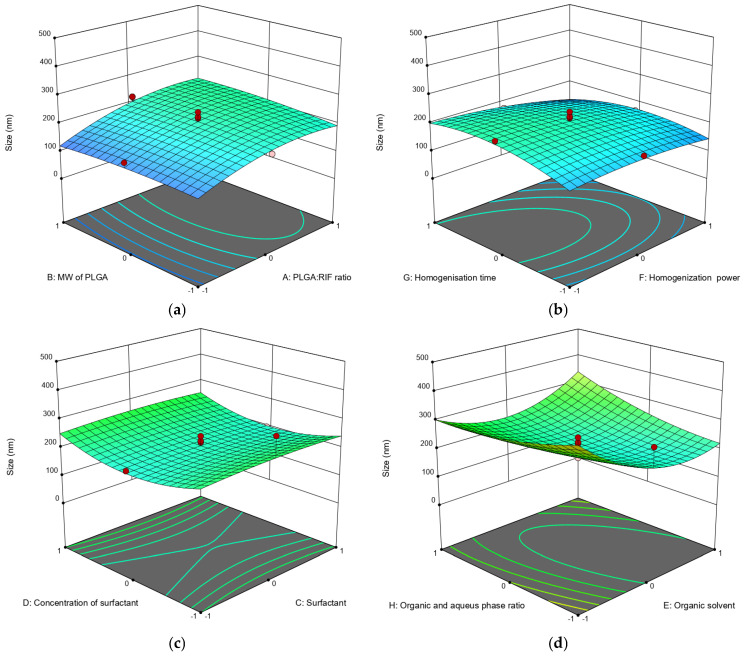
Three-dimensional (3D) response surface diagrams of the impact of independent factors on average particle size: (**a**) MW of PLGA—PLGA:RIF ratio; (**b**) homogenization power—homogenization time; (**c**) surfactant concentration—surfactant; (**d**) organic phase:aqueous phase ratio—organic solvent.

**Figure 2:**
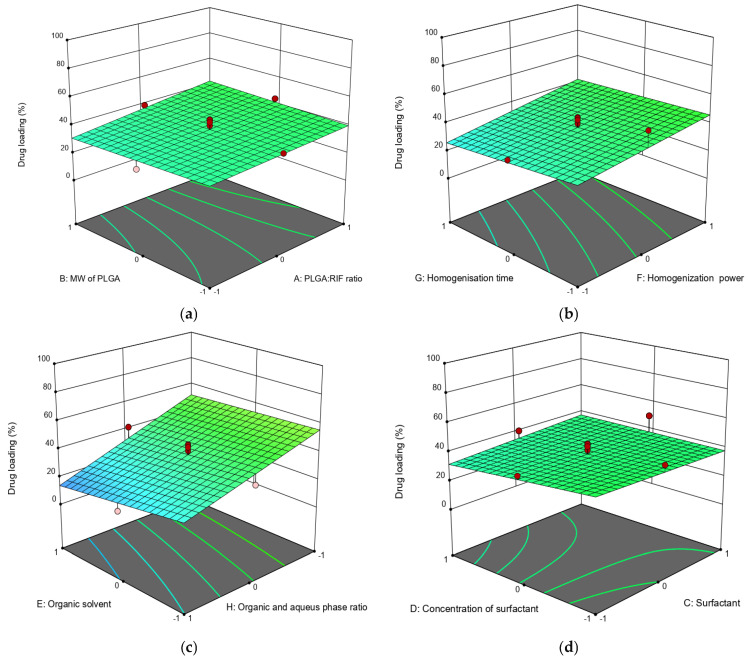
Three-dimensional (3D) response surface diagrams of the impact of independent factors on drug loading: (**a**) MW of PLGA—PLGA:RIF ratio; (**b**) homogenization power—homogenization time; (**c**) organic phase:aqueous phase ratio—organic solvent; (**d**) surfactant concentration—surfactant.

The effects of various factors on nanoparticle size and drug loading rate are depicted using three-dimensional (3D) diagram (Figure 1 and Figure 2).

Contrary to the data previously predicted via the calculations [31], we experimentally found that there is no significant effect of PLGA molecular weight on the sizes of nanoparticles. This parameter is more affected by drug concentration and higher PLGA:RIF ratios. The larger particle size (Figure 1a) is explained by the inclusion of the drug in the space of polymeric NPs. The influence of ultrasonic homogenization on the parameters of the process of producing PLGA NPs was established for the first time. In particular, the data obtained show that a higher power and longer time of ultrasonic homogenization (70 W and 15 min) result in smaller PLGA particle sizes (up to 138 nm).

We also found that any of the used surfactants (PVA, Tween 80, Pluronic F127) can produce PLGA nanoparticles of a given size if the concentration and organic phase are properly selected. At the same time, slightly better particle size and zeta potential values were obtained using PVA (Figure 1c and Table 2). Also, optimal dispersibility values were produced when DMSO was used as the organic phase in a 1:5 ratio with the aqueous phase when compared to the use of other organic solvents (Figure 1d).

Apart from the physicochemical parameters of polymer nanoparticles, the drug loading efficiency is important to the polymer. Drug loading efficiency in PLGA nanoparticles depends on its concentration in the system; it can be seen that the degree of drug loading increases with an increasing the ratio of PLGA:RIF (Figure 2a). The molecular weight of PLGA does not significantly affect either the particle size or the loading efficiency of rifampicin (Figure 2a).

It should be noted that the loading efficiency of the drug into the polymer is significantly affected by the power of the homogenizer. When increasing the power from 15 W to 70 W, the loading efficiency of rifampicin increased from 30% to 46% (Figure 2b). This is possibly due to an increase in emulsion quality. Increasing the duration of ultrasonic homogenization in this case does not significantly affect the drug loading efficiency (changes not more than 5%). With an organic to aqueous phase ratio of 1:1 and using dichloromethane as solvent, the loading degree was 63%. On the contrary, the loading efficiency of rifampicin was only 6% when the organic to aqueous phase ratio was 1:10 and ethyl acetate was used (Figure 2c). Regarding the effect of surfactant type and concentration, the rifampicin loading efficiency decreased from 49% to 30% when the surfactant concentration was increased from 0.5% to 1.5%. Nanoparticles had a higher loading efficiency when Pluronic F127 was used as the surfactant (Figure 2d).

Using Design Expert software, the parameters for computer-aided optimization were selected. Table 4 shows which criteria were used for optimization to obtain PLGA-RIF NPs with minimum size and maximum drug loading efficiency.

Based on the results produced by ANOVA, Design Expert software suggested the best parameters for producing PLGA-RIF nanoparticles with minimum size and maximum loading efficiency. The optimum parameters for producing PLGA-RIF nanoparticles were determined as follows: PLGA:RIF ratio—1:1, MW of PLGA—30,000–60,000, surfactant—PVA, organic solvent—DCM, homogenization power—70 W, homogenization time—15 min, organic and aqueous phase ratio = 1:1. The synthesis of PLGA NPs with the proposed parameters produced by the CCD approach was carried out to compare the predicted data with the experimental data. A significant agreement between the predicted and the experimental data was observed (Table 5). Consequently, the CCD method has been found to be effective for the optimization of the PLGA-RIF NPs synthesis process.

### 3.2. Physicochemical Characterization of PLGA-RIF Nanoparticles

**Figure 3:**
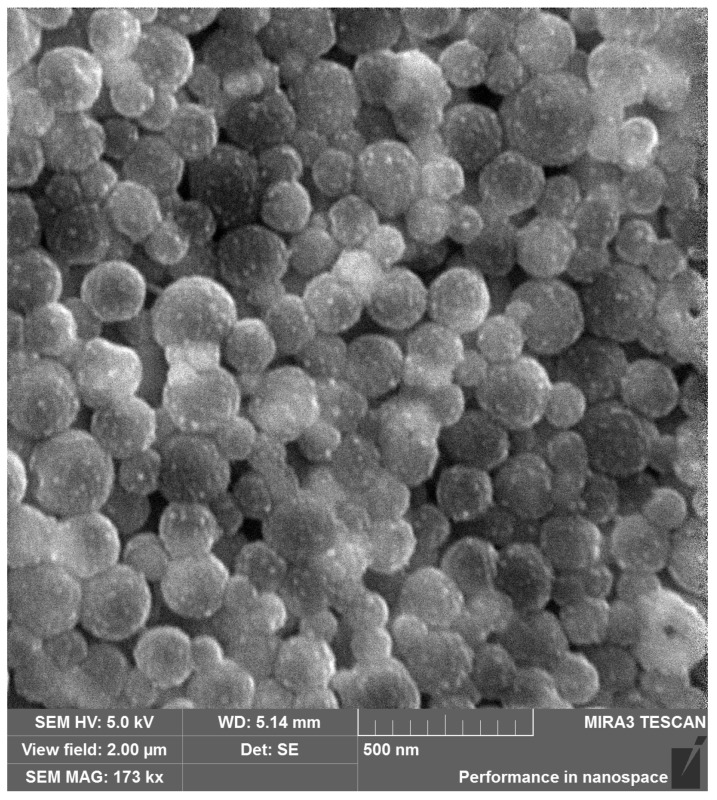
Scanning electron microscopy image of PLGA NPs loaded with rifampicin.

To further analyze the particle size and determine the morphology of PLGA-RIF nanoparticles produced under optimal conditions, scanning electron microscopy was used (Figure 3). The images show that the nanoparticles have a predominantly regular spherical shape with the size not exceeding 200 nm, and they are relatively homogeneous. The significant aggregation of particles is also not observed, indicating that they are quite pliable despite various mechanical effects, including centrifugation during sample cleaning.

**Figure 4:**
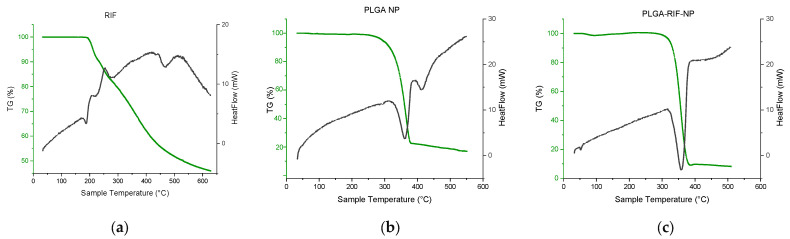
Thermogravimetric analysis and differential scanning calorimetry of: (**a**) Rifampicin, (**b**) PLGA NPs, (**c**) PLGA-RIF NPs.

Furthermore, a number of thermochemical studies were carried out to exclude possible chemical interaction between the polymer and a drug, leading to changes in the biological activity of the drug. Thermogravimetric analysis (TGA) and differential scanning calorimetry (DSC) curves of rifampicin, PLGA NPs and PLGA-RIF NPs are presented in Figure 4.

The DSC curve of rifampicin shows several peaks, which reflect physicochemical changes in the substance upon heating. The first endothermic peak recorded at 186 °C corresponds to the melting point of rifampicin and the transition of the substance from the solid phase to the liquid phase [34]. The next exothermic peak recorded at 196 °C is probably related to the process of recrystallization and change in the polymorphic form. Part of the heat released during recrystallization triggers the decomposition process with a mass loss of 10% [35]. This is followed by an exothermic peak at 252 °C, which is accompanied by another 10% mass loss, corresponding to the second stage of rifampicin decomposition. Then follows the third stage, where the last peak at 422 °C may indicate further changes in the chemical structure of rifampicin and subsequent mass loss, which presumably corresponds to the second stage of decomposition [34,35].

Two endothermic peaks can be observed on the curve that corresponds to placebo PLGA nanoparticles. The first peak observed at 360 °C may reflect the beginning of the thermal decomposition or degradation process of PLGA. Since the percentage of mass loss of the polymer at this point is 77%, this may indicate the onset of significant polymer degradation. The second peak, which appears at 420 °C with slight mass loss, may indicate more complex processes, such as the subsequent degradation of residual polymer parts [6,30,36,37]. As for rifampicin-loaded PLGA nanoparticles, no new endothermic peaks can be observed in the DSC curve. Only one endothermic peak at 357 °C has been detected. The above data prove that there is no chemical interaction between PLGA and rifampicin when the drug is loaded into polymer nanoparticles [6,30].

**Figure 5:**
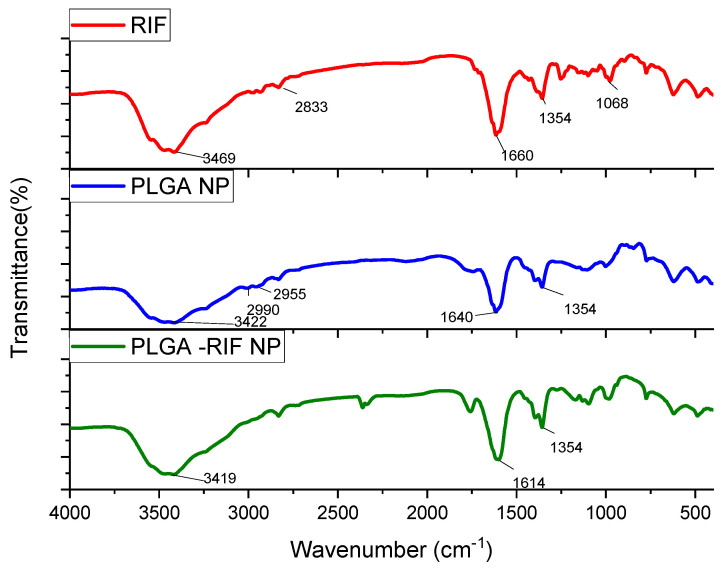
IR spectra for rifampicin, PLGA-NPs and PLGA-RIF NPs.

Another equally informative method for identifying the chemical composition of substances is spectroscopy. The comparison of FTIR spectra of PLGA-RIF NPs with individual components can be seen in the figure below (Figure 5).

Characteristic absorption patterns of rifampicin include distinct peaks at certain frequencies. Strong absorption at 3469 cm^−1^ corresponds to the stretching of the N–H bond. The peak at 2833 cm^−1^ indicates the presence of a C–H bond. The peak recorded at 1660 cm^−1^ represents the C=O group. The peak observed at 1354 cm^−1^ is associated with both CH_2_ and C=C. The absorption band observed at 1068 cm^−1^ usually corresponds to –CH, CO, and C–H [32,38,39,40].

From the analysis of PLGA spectra, the following characteristic signals were identified: the peak at 3422 cm^−1^ is associated with OH stretching, that at 1640 cm^−1^ corresponds to the stretching vibration of the carbonyl C=O, the peaks at 2955 cm^−1^ and 2990 cm^−1^ represent the vibrations of CH_2_ and CH_3,_ respectively, and the peak at 1354 cm^−1^ is due to the bending of CH [41,42]. The spectrum of PLGA-RIF NPs shows distinct peaks characteristic of both the polymer and rifampicin structure, indicating that there is no chemical interaction between PLGA NPs and rifampicin.

### 3.3. In Vitro Release Profile of PLGA-RIF NPs

The investigation of rifampicin’s release from PLGA nanoparticles represents an important aspect of the study, since the determination of the release efficiency of the drug has a direct bearing on its potential therapeutic efficacy. For this purpose, two methods were used for the first time in this study: the Flow-Through Cell method on a CE 7Smart apparatus and the vertical diffusion method on a Franz PHOENIX™ DB-6 cell. Both methods have their advantages, and can further validate the results of the study and provide a more complete understanding of rifampicin release processes from PLGA nanoparticles. Despite the difference in the methods used, the results of rifampicin release matched by 98%, which confirms the reliability of the study and the suitability of the chosen methods to the study objectives.

Different pH values have been used to study the drug release: pH 1.2 is used to simulate conditions in the stomach, which allows the possible behavior of the drug during oral administration and its stability in gastric juice to be assessed. A pH 6.8 represents the conditions in the small intestine, which is also important to evaluations of drug release. A pH value of 7.4 mimics the physiological condition in plasma, which is important for understanding how the drug will behave in the systemic bloodstream after absorption. Although pH 1.2 is not used in the inhalation route of administration, we have included it for a more comprehensive understanding of drug release in different physiological media and possible routes of drug entry into the body.

From the obtained Flow-Through Cell method data, it can be seen that the release of rifampicin from the polymer matrix is most efficient under simulated blood plasma conditions (pH 7.4), where a release of approximately 60% in 48 h was achieved. In comparison, under conditions simulating gastric (pH 1.2) and intestinal (pH 6.8) medium, rifampicin release was significantly less efficient (rifampicin release was approximately 1.5% and 23%, respectively), which may be due to the low drug solubility or changes in polymer properties at different pH [43]. Based on the obtained data, it can be concluded that pH has a significant effect on drug release, and the use of an injectable dosage form of the drug, which provides for the direct administration of the drug into the bloodstream, is the preferred option. This method of delivery ensures optimal blood levels of the drug, and it can maximize the therapeutic efficacy with minimal side effects.

**Figure 6:**
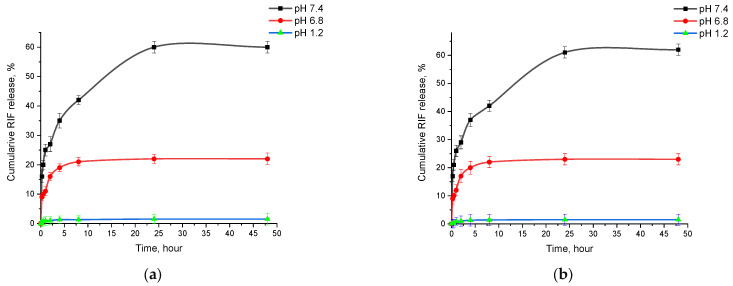
Cumulative release of rifampicin from PLGA-RIF nanoparticles: (**a**) Sotax cell; (**b**) Franz cell.

The study of rifampicin release from optimized PLGA-NPs showed a stable process. Drug release is characterized by an initial burst release followed by a slow or sustained release. The initial rapid release of RIF may be due to the release from the surface of PLGA-NPs, after which the drug is gradually released from the core of nanoparticles due to the hydration and swelling of PLGA NPs [44]. The RIF release study showed the highest release of 62 ± 3.8%, with the phenomenon of sustained release from PLGA-RIF NPs after 24 h (Figure 6b). This process is important to ensuring the controlled and gradual release of the drug, which may be critical for its therapeutic efficacy [45].

### 3.4. In Vitro Mucoadhesion of PLGA-RIF NPs

**Figure 7:**
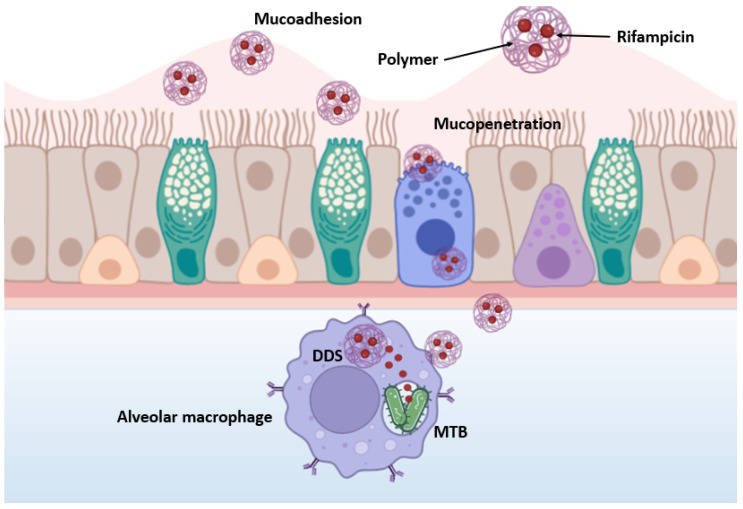
Schematic representation of mucoadhesion and mucopenetrative processes of nanoparticles.

The study of the mucoadhesive properties of nanoparticles is of key importance for the development of effective dosage forms. Figure 7 schematically depicts the mucoadhesion and mucopenetrative processes of NPs during inhaled drug delivery against MTB.

The developed nanoparticles are expected to cross several biological barriers, including the trachea, bronchioles, mucociliary clearance, granulomas, infected cell membranes and MTB biofilm [46]. In the beginning, polymeric particles interact tightly with mucin, resulting in their contact, wetting and swelling. Then, physicochemical interactions occur that promote the adsorption and molecular penetration of the polymer chains into the mucosal layer covering the epithelial surface, ultimately leading to mucopenetration [47]. After overcoming the previous barriers, alveolar macrophages and dendritic cells readily absorb the NPs and the encapsulated drugs are released. Thus, nanoparticles provide controlled and prolonged drug release and allow for increased drug bioavailability. Unlike free drugs, which often do not penetrate well into infected macrophages, nanocarriers can act as “Trojan horses” by carrying drugs inside cells, providing a more effective treatment for TB [46].

**Figure 8:**
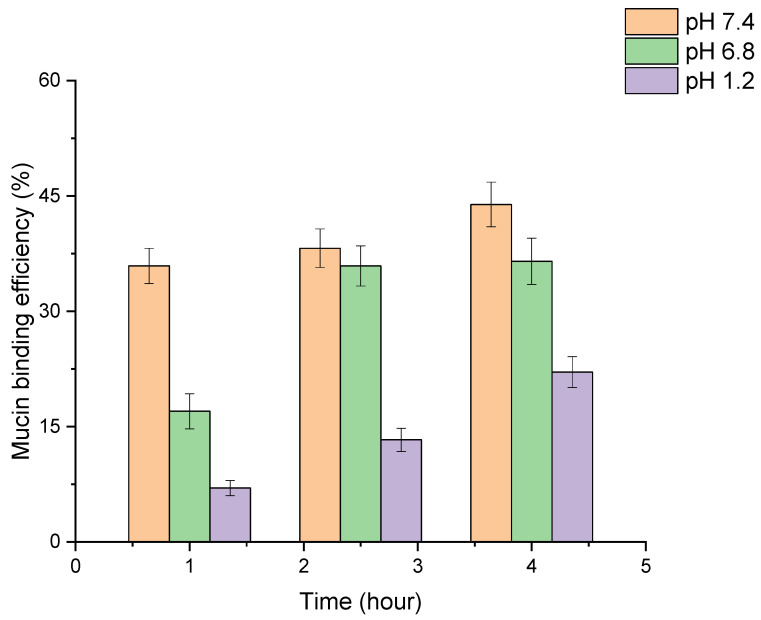
Investigation of mucoadhesive properties of PLGA-RIF nanoparticles.

Mucoadhesive nanoparticles can increase the contact time of the drug with mucosal sites, which improves the bioavailability, targeted delivery and controlled release of active ingredients. This is particularly important for drugs that need to be delivered to specific parts of the gastrointestinal (GIT) tract [48,49,50,51,52]. The results of the study of the adhesion of PLGA-RIF NPs to mucin are shown in Figure 8.

In the study of mucoadhesive properties of PLGA nanoparticles, the turbidity of NP–mucin dispersion was evaluated at different pH levels simulating the GIT medium. An increase in the conjugation of mucin to the nanoparticle surface was observed over time in all three media. The highest rate of binding of mucin to the nanoparticles occurred at pH 7.4, where the efficiency reached 44% after 4 h. At pH 6.8, the binding efficiency was 37% over the same period, showing a more rapid attainment of stable levels. The lowest binding was observed at pH 1.2, with a maximum efficiency of 22% after 4 h. Therefore, we can see a high degree of adhesion of PLGA-RIF NPs to mucin and the dependence of this factor on the environmental pH, which can be used in modeling dosage forms.

### 3.5. In Vitro Efficacy of PLGA-RIF NPs against Strain H37Rv

As part of the MBT studies, all work was conducted in certified Biosafety Level 3 (BSL-3) facilities. Studies were performed in Airstream AC2-4E8 biosafety cabinets (Esco Micro Pte. Ltd., Singapore), which provided sterile conditions through negative pressure and air filtration through HEPA filters. Studies of the antituberculosis activity of nanoparticles were carried out on antituberculosis-sensitive wild strain MTB H37Rv produced by the pulmonology clinic of Asklepios Gauting (Germany). The determination of the bacteriostatic activity of nanoparticles in vitro was evaluated by the growth of the MTB strain on dense Lowenstein–Jensen nutrient medium.

**Figure 9:**
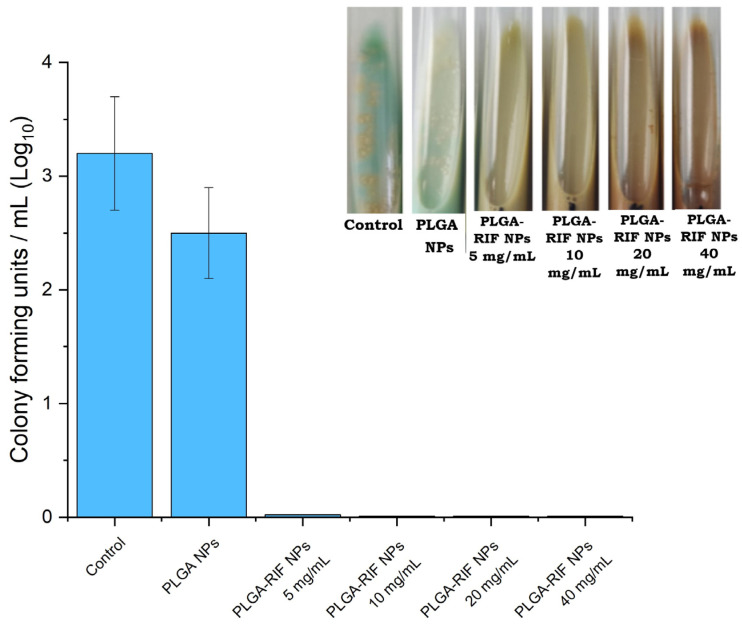
In vitro mycobacterial activity of PLGA-RIF nanoparticles.

To study the mycobacterial activity of the nanoparticles, PLGA nanoparticles were synthesized without the drug as well as encapsulated with rifampicin under optimized conditions. The PLGA-RIF NPs were weighed so that the concentration of rifampicin in the NPs was 5, 10, 20 and 40 mg/mL. The culture tubes were incubated for 4 weeks and after that the results were recorded. The data showing the effects on the growth of wild strain MTB H37Rv are presented in Figure 9.

After the incubation of empty PLGA nanoparticles at 37 °C, no antimicrobial activity was demonstrated. In particular, PLGA nanoparticles without drugs had no effect on inhibiting the growth of the Mycobacterium tuberculosis H37Rv strain. This indicates that PLGA nanoparticles alone do not have antimicrobial properties and do not affect the growth of mycobacteria without the addition of active ingredients. PLGA-RIF NPs were sensitive to mycobacteria and completely inhibited the growth of the H37Rv strain. Since the polymeric matrix of the nanoparticles provides the prolonged release of rifampicin, it maintains therapeutic concentrations of the drug for a long time, which is important for the complete eradication of slow-growing and latent forms of MTB. Based on the results obtained on mycobacterial activity, there is hope for the further application of polymeric NPs with antituberculosis drugs for the treatment of tuberculosis. Thus, the use of NPs with prolonged action will help solve problems such as non-compliance with the dosage and frequency of drug administration. It will also reduce side effects and help in overcoming the multidrug resistance of mycobacteria to RIF.

## 4. Conclusions

The obtained results indicate that the multifactorial optimization of the process for producing rifampicin-loaded polylactide-co-glycolide nanoparticles can be carried out by use of the central composite design method. The optimum parameters for producing PLGA-RIF nanoparticles were determined as follows: PLGA:RIF ratio—1:1, MW of PLGA—30,000–60,000, surfactant—PVA, organic solvent—DCM, homogenization power—70 W, homogenization time—15 min, organic and aqueous phase ratio—1:1. In addition to optimal synthesis conditions, we found that the most suitable method for dispersing PLGA from the perspective of both NPs dispersion characteristics and drug incorporation is using PVA as a surfactant and DCM as an organic phase. PVA helps to stabilize and evenly distribute PLGA nanoparticles in the aqueous phase, preventing their aggregation. For the first time in the present study, the effects of ultrasonic homogenization on the dispersibility of the produced NPs during the emulsification of PLGA in an aqueous organic emulsion are shown.

The results related to drug release from polymeric NPs obtained via the Flow-Through Cell and vertical diffusion methods on Franz cells show that RIF reaches its maximum concentration after 24 h, and remains constant in the system during the next 24 h. Thus, the release profile of PLGA-RIF NPs indicates the possibility of producing a prolonged-dosage form of anti-TB drugs, which is very important in the treatment of tuberculosis, since one of the reasons for the low treatment efficacy is non-compliance with the drug regimen.

Insufficient attention is paid by researchers to the mucoadhesion of dosage forms to tissues. Moreover, there are few such works in relation to polymeric NPs. We have investigated the mucoadhesion of PLGA-RIF NPs with respect to mucin, and shown a sufficiently high retention of polymeric NPs. This allows for the future testing of this system not only in the form of oral administration, but also in the form of inhalation into the respiratory tract. The main limitation of the latter method is the poor dissolution of anti-TB drugs in the lungs. It is shown that this system is viable. RIF is incorporated into polymeric NPs in a chemically unchanged form, and it is able to inhibit the growth of *Mycobacterium tuberculosis*, which is demonstrated by strain H37Rv.

## Figures and Tables

**Table 1:** Experimental factors for PLGA-RIF NPs synthesis and corresponding levels.

Independent Variable	Variable Level		
	Low –1	Center 0	High 1
PLGA:RIF	1:1	2:1	3:1
MW of PLGA	7000–17,000	24,000–38,000	30,000–60,000
Surfactant	PVA	Tween 80	Pluronic F127
Concentration of surfactant	0.5%	1%	2%
Organic solvent	DCM	DMSO	EA
Organic phase: aqueous phase	1:1	1:5	1:10
Homogenization power	15 W	35 W	70 W
Homogenization time	5 min	10 min	15 min

**Table 2:** Development of PLGA-RIF nanoparticle formulations for central composite design and the parameters used for their evaluation.

NPs	PLGA:RIF Ratio	MW PLGA	Surfactant	Surfactant Concentration, %	Organic Solvent	Organic Phase:Aqueous Phase	Homogenization Power, W	Homogenization Time, min	Average Size, nm	PDI	Zeta Potential, mV	LE, %	NPs Yield, %
1	2	3	4	5	6	7	8	9	10	11	12	13	14
NP1	3:1	PLGA30	PVA	0.5	EA	1:1	15	5	189 ± 2	0.046 ± 0.003	−30.9 ± 3.4	58	75
NP2	3:1	PLGA30	PVA	2	DCM	1:1	15	5	422 ± 2	0.280 ± 0.013	−13.1 ± 1.8	64	59
NP3	3:1	PLGA7	Pluronic F127	2	EA	1:10	15	5	316 ± 2	0.349 ± 0.051	−28.2 ± 3.3	24	22
NP4	2:1	PLGA24	Tween 80	1	DMSO	1:5	35	10	220 ± 2	0.177 ± 0.023	−20.1 ± 0.6	45	33
NP5	3:1	PLGA7	Pluronic F127	2	EA	1:1	70	5	94 ± 1	0.285 ± 0.067	−22.8 ± 2.3	88	26
NP6	1:1	PLGA7	PVA	0.5	EA	1:1	70	15	219 ± 2	0.489 ± 0.035	−0.61 ± 2.1	78	41
NP7	2:1	PLGA24	Tween 80	1	DMSO	1:5	35	15	175 ± 2	0.191 ± 0.046	−15.6 ± 3.7	29	67
NP8	3:1	PLGA30	PVA	2	EA	1:10	15	15	452 ± 3	0.499 ± 0.086	−15.8 ± 2.9	31	66
NP9	2:1	PLGA24	Tween 80	1	DMSO	1:5	35	10	170 ± 2	0.358 ± 0.008	−23.2 ± 4.2	40	34
NP10	2:1	PLGA24	Tween 80	1	DMSO	1:5	35	10	172 ± 2	0.178 ± 0.008	−21.6 ± 2.8	37	37
NP11	3:1	PLGA7	PVA	0.5	DCM	1:1	15	5	443 ± 3	0.261 ± 0.034	−14.5 ± 3.4	52	69
NP12	1:1	PLGA30	Pluronic F127	0.5	DCM	1:10	70	5	260 ± 2	0.262 ± 0.045	−24.3 ± 2.2	30	33
NP13	2:1	PLGA24	Tween 80	1	DMSO	1:5	35	10	184 ± 2	0.186 ± 0.080	−23.2 ± 2.1	33	31
NP14	3:1	PLGA7	Pluronic F127	2	DCM	1:10	70	5	219 ± 2	0.248 ± 0.065	−20.3 ± 3.1	23	36
NP15	1:1	PLGA7	PVA	2	EA	1:1	15	5	119 ± 2	0.217 ± 0.013	−11.2 ± 2.7	44	41
NP16	1:1	PLGA7	Pluronic F127	2	EA	1:1	15	15	166 ± 3	0.556 ± 0.039	−20.1 ± 1.4	16	3
NP17	3:1	PLGA7	PVA	0.5	EA	1:10	70	5	356 ± 3	0.474 ± 0.023	−12.8 ± 1.3	48	68
NP18	2:1	PLGA24	Tween 80	1	DMSO	1:1	35	10	291 ± 2	0.373 ± 0.034	−15.4 ± 0.3	37	55
NP19	2:1	PLGA24	PVA	1	DMSO	1:5	35	10	206 ± 3	0.135 ± 0.023	−13.2 ± 1.3	39	33
NP20	1:1	PLGA30	Pluronic F127	2	DCM	1:10	15	15	271 ± 1	0.508 ± 0.056	−9.5 ± 1.5	27	31
NP21	1:1	PLGA30	PVA	0.5	DCM	1:1	70	5	282 ± 2	0.145 ± 0.034	−16.4 ± 2.3	65	61
NP22	2:1	PLGA24	Tween 80	2	DMSO	1:5	35	10	193 ± 4	0.224 ± 0.006	−18.5 ± 3.3	40	28
NP23	2:1	PLGA24	Tween 80	1	DMSO	1:5	70	10	167 ± 3	0.276 ± 0.013	−15.6 ± 1.3	41	17
NP24	1:1	PLGA7	Pluronic F127	0.5	EA	1:1	15	5	94 ± 2	0.206 ± 0.043	−13.9 ± 1.6	48	32
NP25	1:1	PLGA30	Pluronic F127	0.5	EA	1:1	70	15	95 ± 3	0.238 ± 0.023	−20.7 ± 3.1	55	30
NP26	3:1	PLGA7	Pluronic F127	0.5	DCM	1:1	70	15	271 ± 4	0.116 ± 0.032	−17.5 ± 0.7	53	65
NP27	3:1	PLGA7	Pluronic F127	2	EA	1:10	70	15	377 ± 4	0.393 ± 0.051	−23.9 ± 1.9	31	35
NP28	2:1	PLGA24	Tween 80	1	EA	1:5	35	10	225 ± 2	0.269 ± 0.042	−17.8 ± 1.7	41	49
NP29	2:1	PLGA7	Tween 80	1	DMSO	1:5	35	10	182 ± 3	0.157 ± 0.023	−15.7 ± 2.3	38	30
NP30	1:1	PLGA7	PVA	2	DCM	1:10	15	15	165 ± 4	0.263 ± 0.015	−12.5 ± 0.4	4	6
NP31	1:1	PLGA7	Pluronic F127	2	EA	1:10	70	5	345 ± 3	0.393 ± 0.045	−12.1 ± 1.1	13	28
NP32	3:1	PLGA24	Tween 80	1	DMSO	1:5	35	10	202 ± 2	0.179 ± 0.035	−22.6 ± 3.9	44	37
NP33	1:1	PLGA7	Pluronic F127	0.5	DCM	1:10	15	15	232 ± 2	0.198 ± 0.024	−23.5 ± 1.2	14	26
NP34	3:1	PLGA30	PVA	0.5	EA	1:1	70	15	173 ± 3	0.118 ± 0.021	−9.2 ± 1.1	36	57
NP35	1:1	PLGA30	Pluronic F127	2	EA	1:1	15	5	93 ± 2	0.275 ± 0.053	−7.9 ± 1.4	24	17
NP36	1:1	PLGA24	Tween 80	1	DMSO	1:5	35	10	152 ± 3	0.235 ± 0.014	−16.7 ± 1.2	27	9
NP37	3:1	PLGA7	PVA	0.5	EA	1:10	15	15	239 ± 2	0.164 ± 0.032	−9.1 ± 0.3	11	65
NP38	2:1	PLGA24	Tween 80	1	DMSO	1:10	35	10	178 ± 2	0.226 ± 0.035	−13.3 ± 2.5	15	29
NP39	1:1	PLGA30	PVA	2	DCM	1:10	70	5	221 ± 2	0.164 ± 0.003	−13.5 ± 1.9	8	29
NP40	1:1	PLGA30	PVA	2	EA	1:1	70	15	118 ± 3	0.187 ± 0.013	−8.1 ± 0.9	35	25
NP41	3:1	PLGA7	Pluronic F127	2	DCM	1:10	15	15	418 ± 5	0.468 ± 0.022	−11.4 ± 4.1	32	38
NP42	3:1	PLGA30	Pluronic F127	0.5	DCM	1:1	15	5	337 ± 4	0.144 ± 0.006	−21.3 ± 1.1	37	55
NP43	2:1	PLGA24	Tween 80	1	DMSO	1:5	35	10	242 ± 2	0.238 ± 0.011	−20.1 ± 0.9	43	44
NP44	3:1	PLGA30	Pluronic F127	2	EA	1:10	70	5	315 ± 3	0.473 ± 0.018	−16.4 ± 0.4	35	36
NP45	2:1	PLGA24	Tween 80	1	DMSO	1:5	35	5	175 ± 2	0.184 ± 0.023	−20.2 ± 0.6	52	23
NP46	3:1	PLGA30	PVA	0.5	DCM	1:10	70	15	216 ± 3	0.155 ± 0.046	−9.7 ± 1.1	36	52
NP47	3:1	PLGA30	Pluronic F127	2	DCM	1:1	70	15	327 ± 2	0.271 ± 0.007	−3.4 ± 1.4	66	35
NP48	1:1	PLGA7	PVA	0.5	DCM	1:10	70	5	162 ± 2	0.288 ± 0.016	−16.7 ± 5.6	30	8
NP49	2:1	PLGA24	Tween 80	1	DMSO	1:5	15	10	225 ± 3	0.205 ± 0.018	−15.7 ± 1.6	32	27
NP50	2:1	PLGA24	Tween 80	1	DCM	1:5	35	10	354 ± 4	0.416 ± 0.023	−19.9 ± 1.5	34	43
NP51	2:1	PLGA30	Tween 80	1	DMSO	1:5	35	10	220 ± 2	0.175 ± 0.018	−24.5 ± 1.1	39	37
NP52	1:1	PLGA30	PVA	0.5	DCM	1:10	15	5	209 ± 3	0.212 ± 0.024	−16.9 ± 0.6	25	49
NP53	2:1	PLGA24	Tween 80	0.5	DMSO	1:5	35	10	323 ± 4	0.248 ± 0.017	−16.1 ± 4.7	45	45
NP54	3:1	PLGA7	PVA	2	DCM	1:1	70	15	302 ± 5	0.166 ± 0.050	−15.0 ± 2.1	34	59
NP55	1:1	PLGA30	PVA	0.5	EA	1:10	70	15	163 ± 3	0.234 ± 0.080	−17.1 ± 1.1	16	22
NP56	1:1	PLGA7	Pluronic F127	2	DCM	1:1	15	5	257 ± 2	0.176 ± 0.020	−17.8 ± 2.1	50	21
NP57	2:1	PLGA24	Pluronic F127	1	DMSO	1:5	35	10	197 ± 4	0.212 ± 0.030	−15.6 ± 4.6	49	55
NP58	3:1	PLGA30	Pluronic F127	0.5	EA	1:10	15	15	373 ± 3	0.479 ± 0.050	−24.5 ± 2.3	6	20
NP59	2:1	PLGA24	Tween 80	1	DMSO	1:5	35	10	225 ± 3	0.207 ± 0.030	−22.4 ± 2.9	44	30
NP60	1:1	PLGA30	PVA	0.5	DCM	1:1	15	15	311 ± 3	0.150 ± 0.050	−18.2 ± 1.6	63	67

PLGA7 is for the poly (D,L-lactide-co-glycolide) (50:50)-ester terminated with a molecular weight 7000–17,000. PLGA24 is for poly (D,L-lactide-co-glycolide) (50:50)-ester terminated with a molecular weight 24,000–38,000.

**Table 3:** ANOVA results for mean NPs size and drug loading efficiency.

Response	Source	Sum of Squares	Degree of Freedom	Mean Square	F-Value	*p*-Value	
Size	Model	4.293 × 10^5^	44	9757.75	3.33	0.0071	significant
	Pure error	4664.39	5	932.88			
	Residual	43,955.54	15	2930.37			
	Lack of fit	39,291.15	10	3929.11	4.21	0.0630	
	Cor total	4.733 × 10^5^	59				
Loading efficiency	Model	15,793.89	36	438.72	8.98	<0.0001	significant
	PureError	100.33	5	20.07			
	Residual	1124.08	23	48.87			
	LackofFit	1023.75	18	56.88	2.83	0.1263	
	CorTotal	16,917.98	59				

**Table 4:** Limitations on independent variables and outcomes.

Name	Goal	Lower Limit	Upper Limit
PLGA:RIF ratio	Is in range	1:1	1:3
MW of PLGA	Is in range	7000–17,000	30,000–60,000
Surfactant	Is in range	PVA	Tween80
Organic solvent	Is equal to DCM	DCM	EA
Homogenization power	Is equal to 70 W	15 W	70 W
Homogenization time	Is in range	5 min	15 min
Organic and aqueous phase ratio	Is equal to 1:1	1:1	1:10
Size	Minimize	93.4	451.8
Loading efficiency	Maximize	4	88.2

**Table 5:** Predicted and experimental results for PLGA-RIF nanoparticles.

	Size, nm	PDI	Zeta Potential, mV	Encapsulation Efficiency, %	Loading Efficiency, %	Yield, %
Predicted	228	0.120	−24	93	70	45
Experimental	223 ± 2	0.110 ± 0.01	−26 ± 2	91 ± 2	67 ± 1	47 ± 2
Error (%)	2.2	9.1	7.7	2.2	4.5	4.3

## Data Availability

The original contributions presented in the study are included in the article and Supplementary Materials, further inquiries can be directed to the corresponding authors.

## Supplementary Materials

The following supporting information can be downloaded at: https://www.mdpi.com/article/10.3390/polym16172466/s1, Figure S1. Contour plots of the best parameters to obtain PLGA-RIF nanoparticles based on (a) particle size, (b) polydispersity index, (c) ζ potential, (d) encapsulation efficiency, (e) loading degree of rifampicin and (f) NPs yield. Figure S2: Calibration curve for rifampicin determined by HPLC method. Figure S3: Calibration curve for rifampicin determined by UV spectroscopy at different pH: (a) pH 1.2; (b) pH 6.86; (c) pH 7.4.
