# Supplementary material for: Optimization of Polylactide-Co-Glycolide-Rifampicin Nanoparticle Synthesis, In Vitro Study of Mucoadhesion and Drug Release
Source: Polymers (Basel). 2024 Aug 30;16(17):2466. doi: 10.3390/polym16172466 (PMC11397862; doi:10.3390/polym16172466)
Supplement: Supplementary file 1 [file polymers-16-02466-s001.zip › polymers-3132423-supplementary.pdf]

OPTIMIZATION OF POLYLACTIDE-CO-GLYCOLIDE- RIFAMPICIN NANOPARTICLE SYNTHESIS, IN VITRO STUDY OF MUCOADHESION AND DRUG RELEASE

Nazgul A. Yessentayeva[1], Aldana R. Galiyeva[1], Arailym T. Daribay[1], Daniyar T. Sadyrbekov[1], Rouslan I. Moustafine[2] and Yerkeblan M. Tazhbayev[1]

[1]Karaganda Buketov University, Karaganda, Kazakhstan
[2]Institute of Pharmacy, Kazan State Medical University, Kazan, Russia
Correspondence: aldana_karaganda@mail.ru, tazhbaev@mail.ru

After analyzing the data using ANOVA, parameters were selected to optimize the process to produce NPs with the minimum size and maximum drug loading (Figure 1).

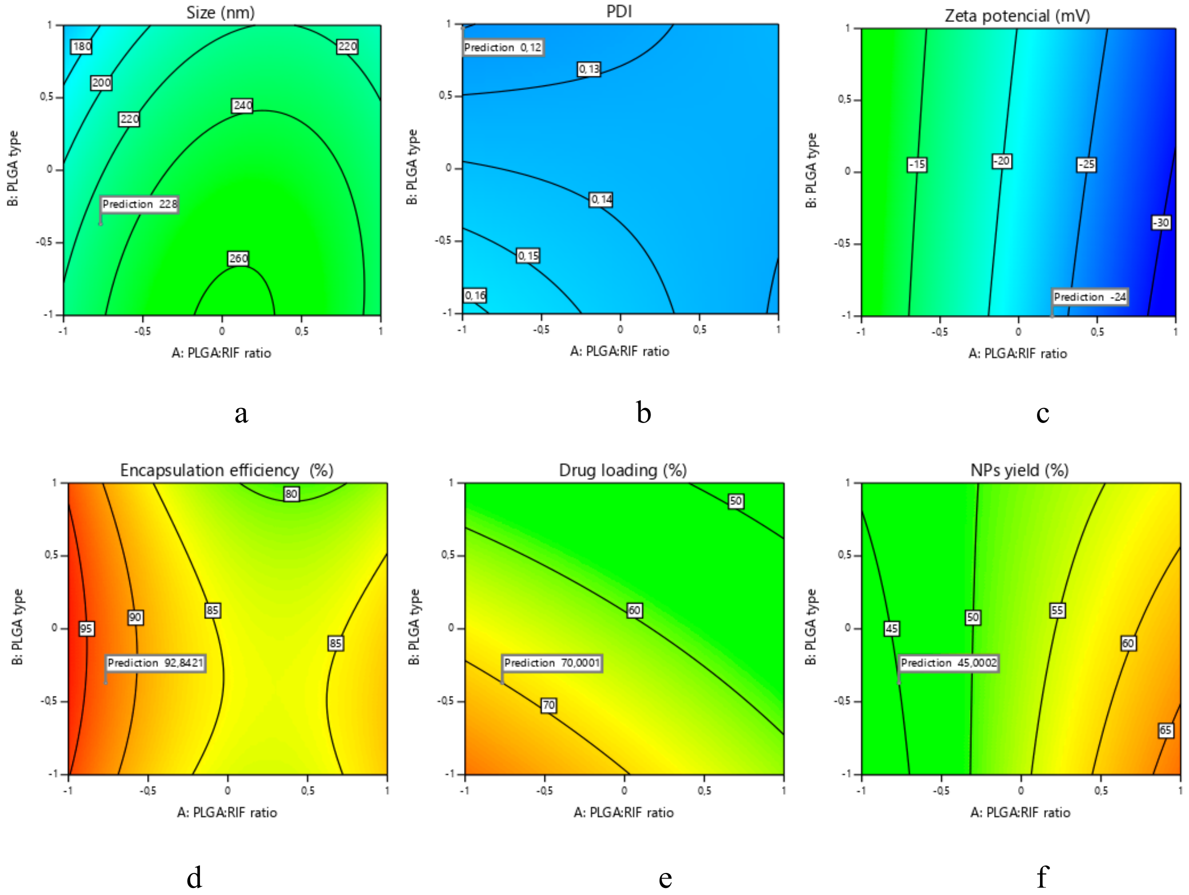

Figure 1. Contour plots of the best parameters to obtain PLGA-RIF nanoparticles based on (a) particle size, (b) polydispersity index, (c) ζ potential, (d) encapsulation efficiency, (e) loading degree of rifampicin and (f) NPs yield.

The linear equation of the calibration curve and linear correlation coefficient for rifampicin determined by HPLC is shown in Figure 2.

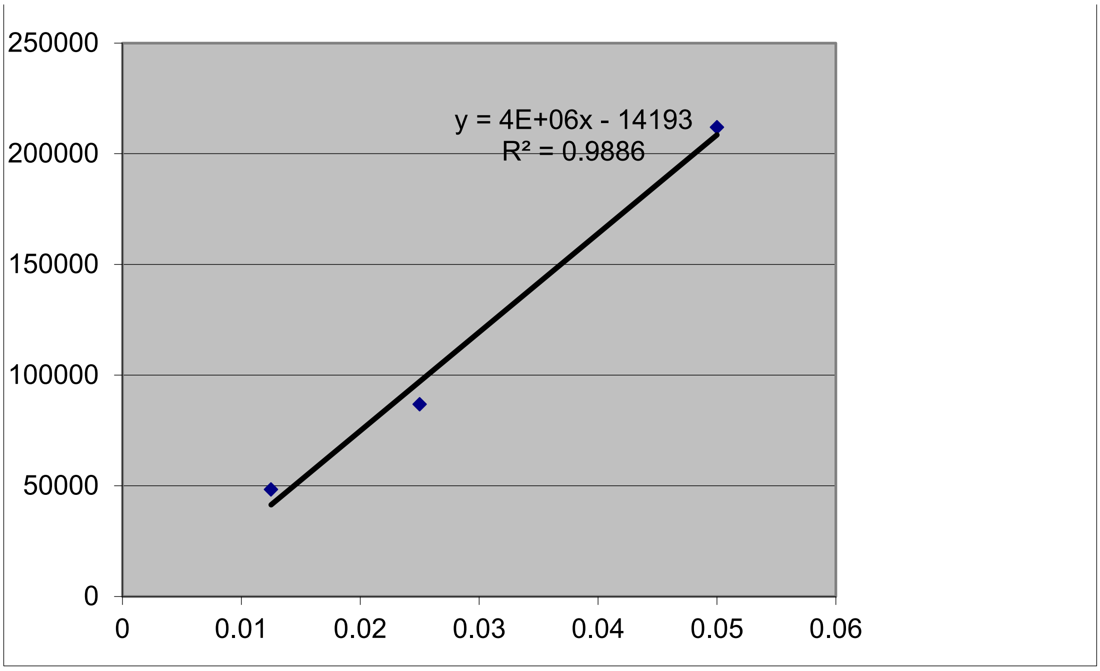

Figure 2: Calibration curve for rifampicin determined by HPLC method

The calibration curve of the standard linear equation and linear correlation coefficient for rifampicin at different pH of the medium determined by UV spectroscopy is shown in Figure 3.

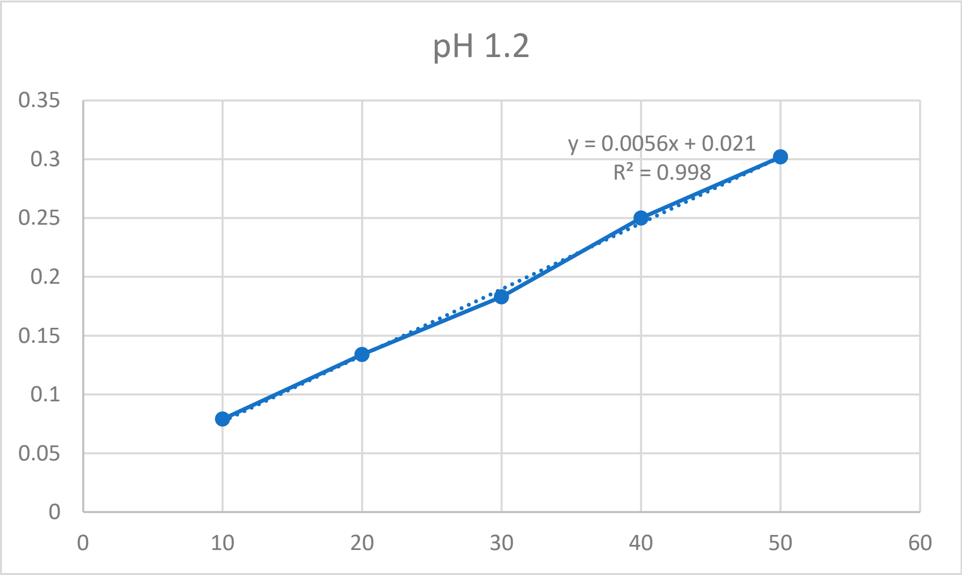

(a)

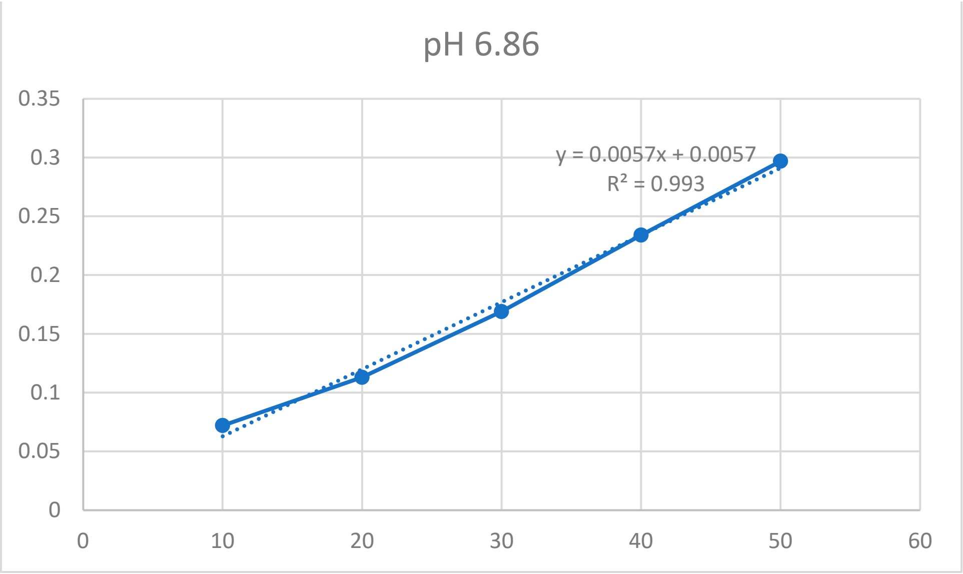

(b)

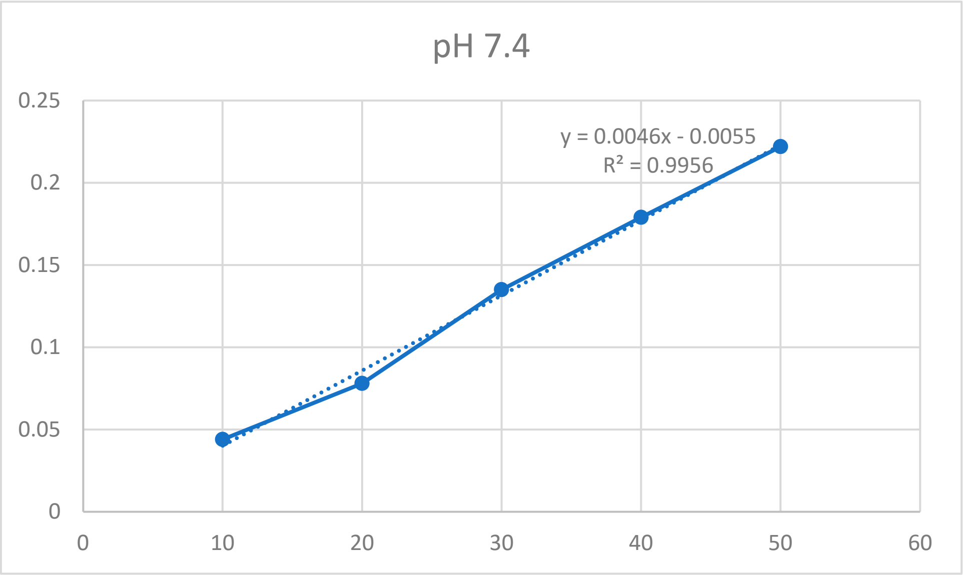

(c)

Figure 3: Calibration curve for rifampicin determined by UV spectroscopy at different pH: (a) pH 1.2; (b) pH 6.86; (c) pH 7.4
